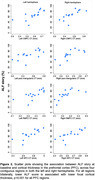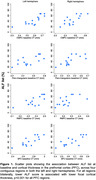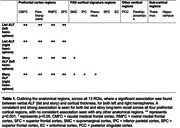# The association between focal prefrontal cortical atrophy and accelerated long‐term forgetting in presymptomatic autosomal dominant Alzheimer’s disease

**DOI:** 10.1002/alz.092404

**Published:** 2025-01-03

**Authors:** Chloe Young, Maggie R Fraser, Kirsty Lu, Antoinette O'Connor, Sebastian J Crutch, Nick C Fox, Philip SJ Weston

**Affiliations:** ^1^ UCL, London United Kingdom; ^2^ Dementia Reseach Centre, Queen Square Institute of Neurology, University College London, London United Kingdom; ^3^ Dementia Research Centre, UCL Queen Square Institute of Neurology, University College London, London United Kingdom; ^4^ Dementia Research Centre, UCL Queen Square Institute of Neurology, London United Kingdom; ^5^ UK Dementia Research Institute at UCL, London United Kingdom

## Abstract

**Background:**

Accelerated long‐term forgetting (ALF) is a form of episodic memory impairment where information is retained normally over 30‐60 minutes but lost at an accelerated rate over subsequent days to weeks, and is a very early – perhaps the earliest – cognitive change in both autosomal dominant and sporadic Alzheimer’s disease (AD). However, the neuroanatomical changes underlying ALF in AD have remained elusive. We explored associations between ALF and focal cortical thickness in presymptomatic autosomal dominant AD (ADAD).

**Method:**

Eighteen asymptomatic ADAD mutation carriers (mean estimated years to symptom onset = 7.3) (SD = 4.4) and twelve non‐carrier controls underwent ALF testing for three tasks (list, story, figure). After learning, recall was tested at 1) 30 minutes and 2) seven days. ALF score was calculated as 7‐day recall/30‐minute recall. T_1_‐weighted MRI was acquired at baseline and annually over five years. Cortical thickness was estimated using FreeSurfer. Thirteen regions of interest were selected based on either a known vulnerability to AD‐related neurodegeneration or a known role in memory. Serum NfL– a neurodegeneration marker– was measured on the single molecule array platform. Spearman coefficients explored associations between ALF and 1) focal baseline cortical thickness, 2) future rates of thinning, and 3) serum NFL.

**Result:**

Across all three tasks, ALF scores were significantly lower in mutation carriers than non‐carriers, despite no difference at 30‐minutes. In mutation carriers, verbal ALF (list and story) was associated with lower baseline cortical thickness in the prefrontal cortex (PFC) across four contiguous regions bilaterally (p<0.001 for each). This association was not present in non‐carriers. No associations were found between ALF and the thickness/volume of medial temporal lobe (MTL) structures. ALF was predictive of future cortical thinning across AD vulnerable regions. Higher serum NFL was associated with poorer long‐term list recall. Forgetting at 30 minutes was not associated with cortical thickness or NfL, i.e. associations were specific to ALF.

**Conclusion:**

ALF is an early presymptomatic marker of AD‐related cognitive decline, underscored by changes in the PFC but not the MTL. These findings advance our understanding of the neuroanatomical substrate of early AD memory decline. ALF could be used as a biologically relevant measure in future presymptomatic trials.